# Incidence of transient interruption of contrast (TIC) – A retrospective single-centre analysis in CT pulmonary angiography exams acquired during inspiratory breath-hold with the breathing command: “Please inspire gently!”

**DOI:** 10.1371/journal.pone.0210473

**Published:** 2019-01-17

**Authors:** Sonja Sudarski, Holger Haubenreisser, Thomas Henzler, Carolin Reischauer, Orpheus Kolokythas, Simon Matoori, Bernhard A. Herzog, Stefan O. Schönberg, Andreas Gutzeit

**Affiliations:** 1 Institute of Clinical Radiology and Nuclear Medicine, University Medical Centre Mannheim, Medical Faculty Mannheim—Heidelberg University, Mannheim, Germany; 2 Institute of Radiology and Nuclear Medicine, Clinical Research Unit, Hirslanden Hospital St. Anna, Luzern, Switzerland; 3 Department of Radiology, University of Washington, Seattle, Washington, United States of America; 4 Department of Chemistry and Applied Biosciences, Institute of Pharmaceutical Sciences, ETH Zurich, Zurich, Switzerland; 5 HerzClinic Luzern, Luzern, Switzerland; 6 Department of Radiology, Paracelsus Medical University, Salzburg, Austria; A.C. Camargo Cancer Center, BRAZIL

## Abstract

**Objectives:**

To assess the occurrence of transient interruption of contrast (TIC) phenomenon in pulmonary computed tomography angiography (CTPA) exams performed in inspiratory breath-hold after patients were told to inspire gently.

**Methods:**

In this retrospective single-centre study, CTPA exams of 225 consecutive patients scanned on a 16-slice CT scanner system were analysed. A-priori to measurements, exams were screened for inadequate pulmonary artery contrast due to incorrect bolus tracking or failure of i.v. contrast administration. Those exams were excluded. Attenuation values in the thoracic aorta and in the pulmonary trunk were assessed in duplicate measurements (M1 and M2) and the aorto-pulmonary density ratio was calculated. An aorto-pulmonary ratio > 1 with still contrast inflow being visible within the superior vena cava was defined as TIC.

**Results:**

3 patients were excluded due to incorrect bolus tracking. Final analysis was performed in 222 patients (mean age 65 ± 19 years, range 18 to 99 years). Mean density in the pulmonary trunk was 275±17 HU, in the aorta 208 ± 15 HU. Mean aorto-pulmonary ratio was 0.81± 0.29. 48 patients (21.6%) had an aorto-pulmonary ratio >1. Correlation of mean aorto-pulmonary ratio and age was: -0.213 (p = 0.001). Age was not significantly different for an aorto-pulmonary ratio >1 vs. ≤1 (p = 0.122). Both in M1 and M2, 33/222 patients presented with absolute HU values of < 200 HU within the pulmonary artery. In M1 measurements, 24 of these 33 patients (72%) fulfilled TIC criteria (M2: 25/33 patients (75%)).

**Conclusions:**

TIC is a common phenomenon in CTPA studies with inspiratory breath-hold commands after patients were told to inspire gently with an incidence of 22% in our retrospective cohort. Occurrence of TIC shows a significant negative correlation with increasing age and disproportionately often occurs in patients with lower absolute contrast density values within their pulmonary arteries.

## Introduction

Computed tomography pulmonary angiography (CTPA) is the international and widely accepted gold standard to investigate patients with suspected pulmonary embolism [1].

CTPA is a non-invasive technique to visualize pulmonary arteries and possible intravascular emboli, and is obtained by the intravenous administration of an iodinated contrast agent.

The diagnostic quality of a CTPA exam is dependent on optimal contrast within the pulmonary arteries (PA).

In the literature, there is consensus that patients could have suboptimal PA contrast enhancement despite optimized contrast bolus techniques and standardized contrast media administration [2–7]. There has been an on-going debate regarding the influence of breathing and breath-holding on image quality and contrast attenuation in the pulmonary arteries during CTPA [2–6]. In 2007, it was argued that transient interruption of contrast in the pulmonary arteries represents a flow-related phenomenon associated with an increased inferior vena cava (IVC) contribution to the right side of the heart [7]. It has been further suggested that this phenomenon could be induced or worsened by deep inspiration [4–6]. If the patient takes a sudden sharp inspiration a sudden bolus of unopacified blood is sucked into the right atrium (RA) via the (IVC) by the sudden drop in intra-thoracic pressure and the superior vena cava (SVC) collapses as the blood is sucked into the RA [8]. These sudden changes in intrathoracic pressure were seen to be the most likely explanation for reduced contrast in the subsequently acquired CTPA, the so-called “transient interruption of contrast” (TIC) phenomenon [9, 10]. Papers were published that showed that end-expiratory breath-hold commands reduce occurrence of TIC [2, 6]. However, some researchers argue that any changes in pressure by different phases of respiration are overwhelmed by a strained breath-hold in the end, where an increase in intra-thoracic pressure occurs leading to reduced flow of contrast medium down the SVC and therefore suggested that the correct instruction is to ask the patient to take a gentle breath in and hold the breath in inspiration in a relaxed manner in order to minimized sudden changes in intrathoracic pressure [8].

Interruption of the contrast bolus results in low contrast attenuation with reduced image quality and a presumably higher rate of false positive and false negative rates of pulmonary embolism [6].

In the literature, the incidence of TIC ranges between 3% and 37% [3, 6, 7]. In pregnant women with increased intra-abdominal pressure, TIC has been reported to be an even more frequent phenomenon causing up to 80% non-diagnostic pulmonary CTA studies [11].

To the best of our knowledge, no study has been published that has investigated the occurrence of TIC in a larger cohort of patients undergoing CTPA scans performed in inspiratory breath-holding after the patients were told to inspire gently to avoid sudden changes in intrathoracic pressure.

The aim of this study was therefore to retrospectively investigate the incidence of TIC in consecutive CTPA exams acquired in inspiratory breath-hold after the patients were told to solely inspire gently.

## Materials and methods

### Study population

This retrospective data analysis was performed in accordance to Health Insurance Portability and Accountability Act (HIPAA) and the Declaration of Helsinki. Institutional review board approval and the need for written informed consent was waived due to the retrospective nature of the study. The patient data used in your retrospective study were extracted from our PACS (Picture archiving and communication system) where meta data like gender or age of the patient are recorded along with the radiological image data. The data acquired in the course of the data analysis were anonymized before being sent to the statistician for statistical analysis. We analysed the datasets of 225 consecutive patients who underwent clinically indicated CTPA for evaluation of pulmonary embolism.

### Image acquisition

All datasets were acquired on a 16-slice single-source CT scanner system (Siemens SOMATOM Emotion; Siemens Healthineers, Forchheim, Germany). Thoracic scans were acquired in cranio-caudal scan direction. Contrast enhancement was achieved by injecting a total of 80 mL of iodinated contrast material (Imeron 400, Bracco, Milan, Italy) followed by a saline chaser of 30 mL both at 3.5 mL/s applied via a 20-gauge intravenous ante-cubital catheter using a dual-syringe power injector (Stellant D CT Injection System, MEDRAD, INC., Warrendale, PA). Scan initiation was determined by bolus tracking and threshold-based auto-triggering using a dedicated software application (CARE bolus Siemens Healthcare Sector, Forchheim, Germany) with the region of interest (ROI) placed in the pulmonary trunk and a threshold for auto-triggering of 100 HU. Detailed scan and contrast material administration parameters are listed in Table 1. Scanning was performed during inspiratory breath-hold. All patients received a breathing command via an automated voice instructing them explicitly to inspire gently and then to hold their breath.

**Table 1:** Scan protocol and contrast material injection protocol parameters.

CTPA^a^ Protocol	
Scanner type (rows)	Single-source (16)
Collimation	16 x 1.2 mm
Rotation time	0.6 s
Pitch	0.95
Bolus Tracking ROI^b^ location	Pulmonary trunk
Auto-trigger-threshold	100 HU^c^
Monitoring scans (n)	30
Monitoring scans delay	3 s
Monitoring scans cycle time	1.5 s
Delay of scan start after auto-triggering	6 s
Tube voltage	130 kVp^d^
Reference tube current	110 Eff mAs^e^
Tube current modulation	On (Care Dose)
Scan direction	cranio-caudal
i.v. catheter size	20 G^f^
CM^g^–Iodine concentration	400 mg/mL
CM-flow	3.5 mL/s
CM-bolus amount	80 mL
NaCl-flow	3.5 mL/s
NaCl-bolus amount	30 mL/s
IDR^h^	1.4 gI/s

a CTPA = computed tomography pulmonary angiography

b ROI = region of interest

c HU = Hounsfield unit

d kVp = peak kilovoltage

e Eff. mAs = Effective mAs = mAs × exposure time, with exposure time = gantry rotation time/pitch

f G = gauge

g CM = contrast material

h IDR = iodine delivery rate.

### Image reconstruction

CTA raw data were reconstructed using a medium sharp convolution kernel with a slice thickness of 1.5 mm and an increment of 1.3 mm.

### Image analysis

**Fig 1:**
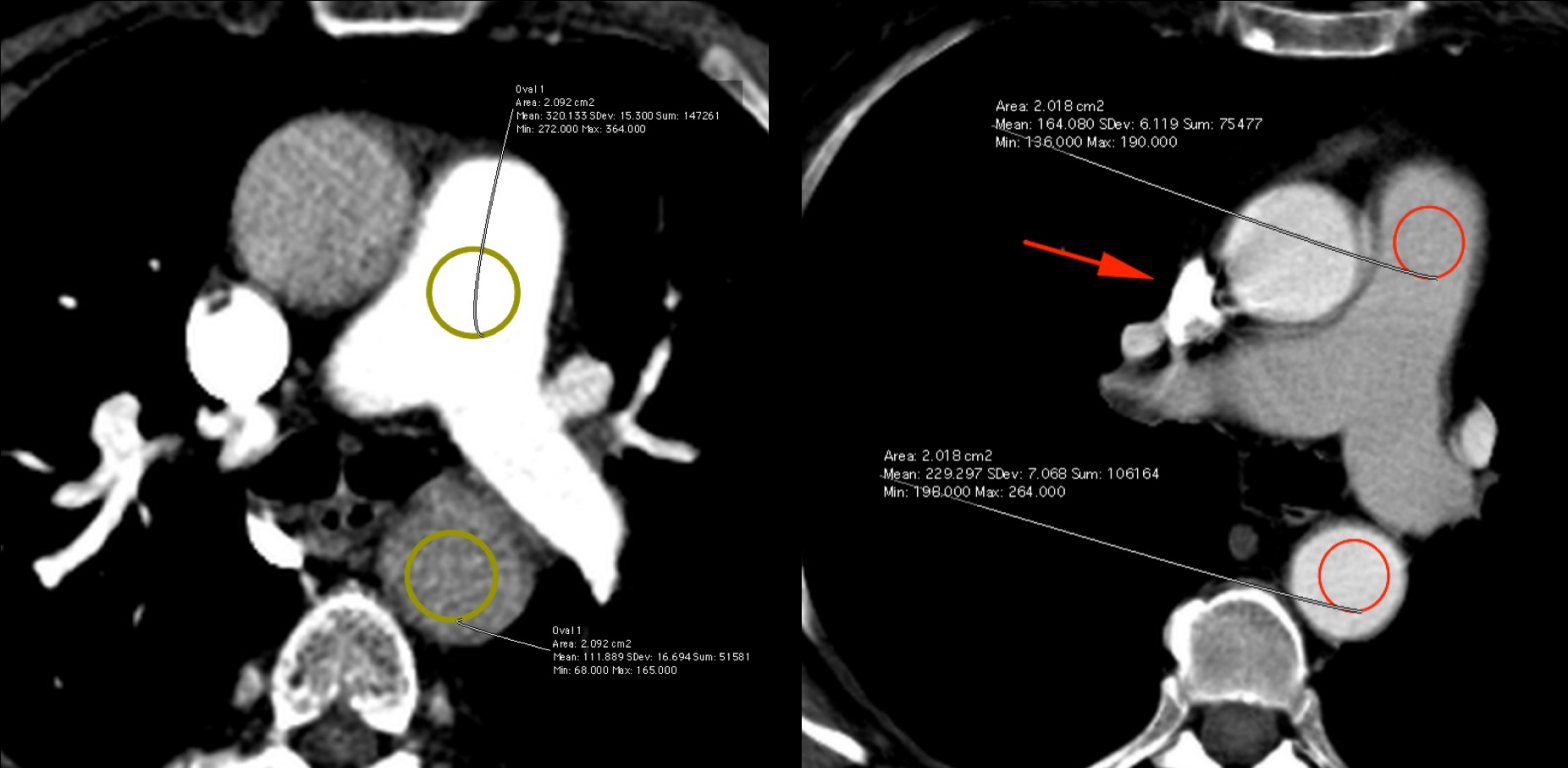
Aorto-pulmonary ratios <1 and >1 –Examples. a) Case of a patient presenting with an aorto-pulmonary ratio < 1. b) Case of a patient with TIC, presenting with an aorto-pulmonary ratio > 1, while contrast material inflow can still be seen within the compressed/collapsed aspect of the *SVC* superior vena cava (SVC) (red arrow).

In consensus reading, two experienced radiologists (blinded for review) with 16 and 5 years of experience in chest CT measured attenuation (Hounsfield units, HU) in duplicate measurements M1 and M2 within the thoracic descending aorta /aortic arch and the pulmonary trunk by positioning a ROI of 2 cm^2^ over the target vessel in regions without beam hardening or other artefacts and without atherosclerotic plaques or thrombo-embolic material (Fig 1A and 1B). We did not perform further HU measurements within the more distal parts (lobar, segmental or subsegmental) of the pulmonary arterial vascular tree in this study.

If the ratio of aortic density/pulmonary artery density, the so-called aorto-pulmonary ratio was ≤ 1 (Fig 1A), the patient was assigned to have a normal CTPA contrast. If the aorto-pulmonary ratio was >1 (Fig 1B) and contrast inflow over the SVC could still be seen (indicating that the insufficient contrast attenuation in the pulmonary trunk was not due to an insufficiently long contrast material bolus), the patient was assigned to have TIC. If the aorto-pulmonary ratio was >1, but no contrast inflow was seen in the SVC, the CTPA-exam was evaluated for incorrect positioning of bolus-tracking ROI in the “Monitoring-Scan” also being archived in our Picture Archiving and Communication System (PACS) along with the diagnostic images. If this was the case, the patient was assigned to the group of patients with insufficient contrast due to reasons other than TIC. If bolus-tracking was adequate, the two readers looked further in the radiology report and in the PACS system whether there was a note explaining the insufficient contrast like contrast extravasation etc. and the patient was assigned to the group of patients with insufficient contrast due to reasons other than TIC as well.

### Statistical analysis

Statistical analysis was performed using SPSS, Version 20 (IBM Corp., Armonk, NY). Graphs were created using Prism Version 7.0d for Mac OS X (GraphPad Software, La Jolla, California, USA, www.graphpad.com).

Frequencies were expressed in absolute numbers and percentages.

Continuous variables were expressed as mean ± standard deviation.

Reliability was assessed by calculating the intraclass correlation coefficient (ICC). with the two-way random average measure for absolute agreement being determined for both measurements in the aorta and the pulmonary arteries.

The statistical analysis finally assessed the number (percentage) of patients with an aorto-pulmonary ratio > 1 and ≤ 1.

Pearson correlation coefficient was assessed to correlate mean aorto-pulmonary ratio and the patients’ age.

Two-sided t-testing for independent samples was performed in order to assess possible differences in age between patients presenting with TIC and patients without TIC. A p-value < 0.05 indicated statistical significance.

## Results

Out of 225 patients, 3 patients were excluded from the analysis as the insufficient contrast enhancement of their CTPA studies was due to incorrect bolus-tracking. There were no patients being excluded due to other reasons for insufficient contrast as e.g. failure of i.v. contrast administration in our retrospective study cohort. Final analysis was performed with CTPA datasets of 222 patients (mean age 65 ± 19 years, range 18 to 99 years) (S1 Dataset). 2/222 patients underwent two CTPA scans within the analysed timespan, however, the scans were not in the form of immediately repeated scans due to insufficient image quality, but with a temporal distance of 13 days and 35 days, respectively. See Table 2 for detailed density measurements of two measurement rounds M1 and M2 in the pulmonary trunk and the aortic arch/descending aorta.

**Table 2:** Density measurements M1 and M2 in the pulmonary trunk and the aortic arch/descending aorta (n = 222 patients).

Results of density measurements within the pulmonary trunk and the aorta				
	PA^a^ M1^b^	PA M2	A^c^ M1	A M2
*Density measurements in HU*^*d*^				
Minimum	118	62	34	37
25% Percentile	230.8	226.8	178	179.8
Median	270	267	210	209
75% Percentile	323.3	317.5	241	243
Maximum	522	504	406	394
Mean	277.1	272.3	208.3	207.4
Standard Deviation	72.86	73.07	55.8	55
Standard Error of Mean	4.89	4.904	3.745	3.691
Lower 95% CI^e^ of mean	267.5	262.6	200.9	200.1
Upper 95% CI of mean	286.8	282	215.7	214.7

^a^PA = pulmonary artery;

^b^M = measurement;

^c^A = aorta;

^d^HU = hounsfield unit;

^e^CI = confidence interval.

**Fig 2:**
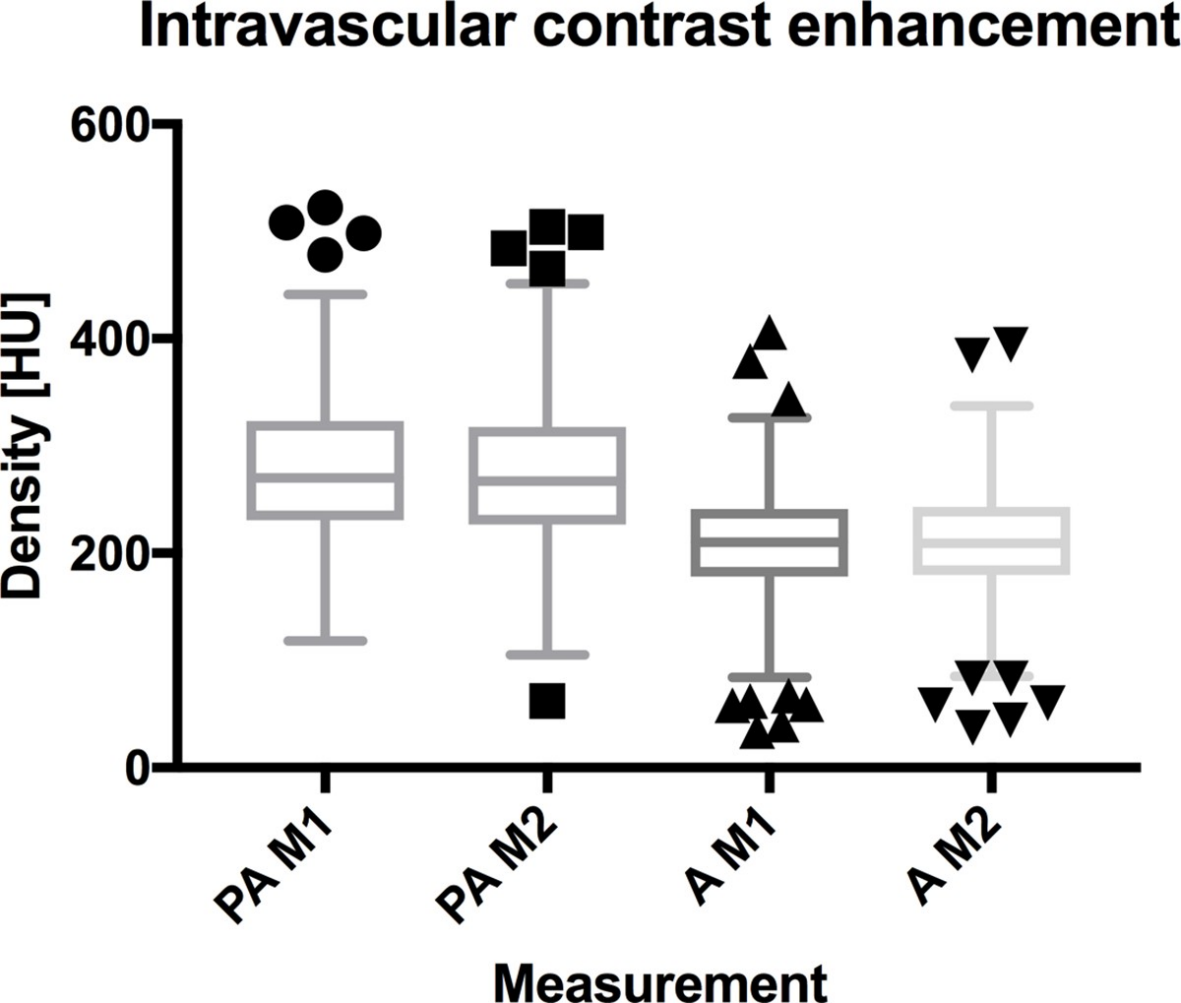
Intravascular contrast enhancement in the pulmonary trunk vs. aorta. Box and whiskers plot (Tukey box plot with the following whiskers’ definition: lowest datum still within 1.5 x IQR of the lower quartile, and the highest datum still within 1.5 x IQR of the upper quartile; values < / > 1.5 x IQR being depicted separately) of intravascular contrast enhancement in the pulmonary trunk and the aortic arch/descending aorta. PA = pulmonary artery; A = aorta; M1 = measurement 1; M2 = measurement 2.

High ICCs were achieved in both the aorta and the pulmonary trunk between duplicate measurements (Fig 2): Reliability of average measures for density of aorta was 0.987 (95% CI, 0.983–0.990). Reliability of average measures for density within the pulmonary trunk was 0.990 (95% CI, 0.985–0.993).

**Fig 3:**
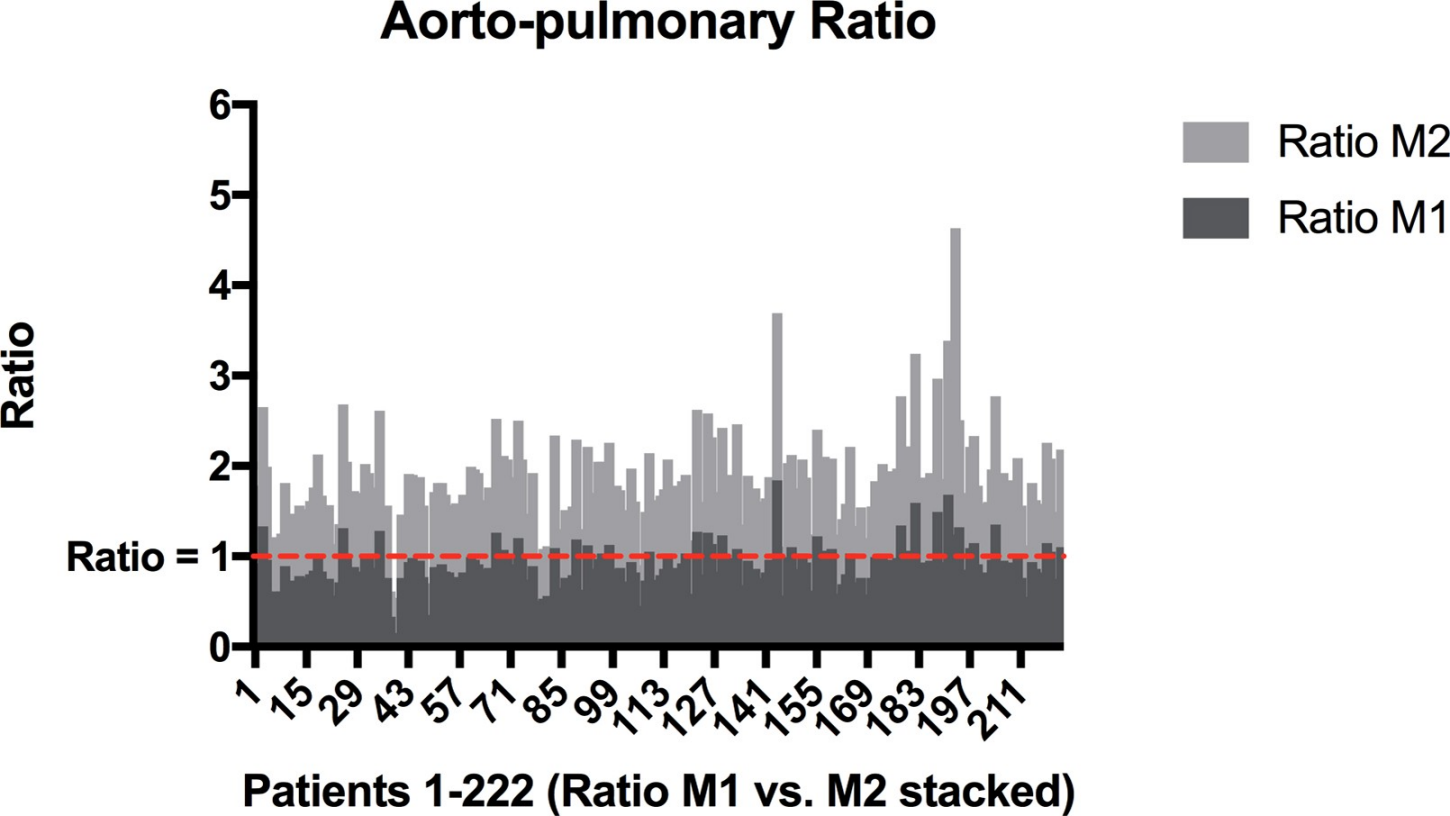
Aorto-pulmonary ratio. Stacked diagram of aorto-pulmonary ratios of measurements M1 (dark grey) and M2 (bright grey) for all patients 1–222; marked with the red grid line is a ratio = 1.

Mean aorto-pulmonary ratio was 0.81 ± 0.29, ranging from 0.13 to 1.84 (25th percentile 0.63, 50th percentile 0.80, and 75th percentile 0.96, respectively). 48 patients (21.6%) had an aorto-pulmonary ratio of >1 while there was still inflow of contrast bolus within SVC, consequently fulfilling our definition of TIC (Fig 3).

Pearson correlation coefficient for correlation of mean aorto-pulmonary ratio and age was: -0.213 (p = 0.001). However, the quotient was >1 also in patients with higher age. Age was not significantly different for an aorto-pulmonary ratio >1 vs. ≤1 (p = 0.122).

Both in M1 and M2, 33/222 patients presented with absolute HU values of < 200 HU within the pulmonary artery, which was markedly below the 25% percentile in both M1 and M2 measurements. According to M1 measurements, 24 of these 33 patients (72%) fulfilled TIC criteria according to our definition, in the second measurement round M2, the same held true for 25/33 patients (75%) fulfilling TIC criteria. Consequently, in the subgroup of patients with absolute HU values ≥200 HU, the percentage of patients fulfilling our TIC criteria were 19/189 (10%) with M1 values and 26/189 (14%) with M2 values.

## Discussion

Our study aimed to evaluate the occurrence of TIC in our retrospective single-centre cohort of patients undergoing clinically indicated CTPA performed during inspiratory breath-holding and patients being explicitly told to inspire gently before holding their breath for evaluation of pulmonary embolism. In our cohort, TIC was a common phenomenon with an incidence rate of 21.6% in all CTPA exams. Absolute HU values of < 200 HU within the pulmonary artery were correlated with a disproportionately higher frequency of TIC (72% and 75% vs. 10% and 14% in measurement rounds 1 and 2 in the subgroup with absolute HU values of 200 and more HU within the pulmonary arteries. This refers to a intermediate to low contrast enhancement according to publications that qualitatively categorized contrast enhancement within the pulmonary trunk [6]. Absolute values > 200 HU were rated as good contrast enhancement in this publication. The aorto-pulmonary ratio shows a significant negative correlation with increasing age, which would be in accordance to the findings in the literature that younger patients tend to inspire more sudden and powerful which is unfavorable with regard to the aorto-pulmonary ratio and the TIC phenomenon.

In the literature, the incidence of TIC ranges between 3% and 37% [3, 6, 7]. However, one has to keep in mind, that different studies have different definitions of TIC: The study by Bernabe-Garcia et al. for example considered TIC to be present when a segment of pulmonary arteries showed lower densities (a difference of at least 10 HU) than other portions of the pulmonary arteries (both proximally and distally) and/or the aorta [12]. Consequently, results varied. In addition, large differences in the incidence of TIC have been reported between inspiratory and expiratory scans: Mortimer et al. reported incidences of 14% vs. 29% in expiratory vs. inspirational scans [6]. In contrast to our study, in this publication, for inspiratory scans the patient was asked to take a breath in and hold before imaging. With gentle inspiration on the other hand, as used in our study, the concept is that the patient reduces the effect of sudden reduction in intra-thoracic pressure causing suction of blood into the right atrium from the extra-thoracic vena cava, the “abdomino-thoracic pump” [6]. However, a second phenomenon that is not influenced by gentle inspiration is forced breath-holding against a closed glottis, a Valsalva manoeuvre. This reduces the venous return within the thorax by increasing in intra-thoracic pressure.

Several studies have compared the impact of different breathing manoeuvres on contrast enhancement in target vessels: In a CTPA-study, Gutzeit et al. compared contrast enhancement within the pulmonary arteries between patients who were scanned during end-inspiratory breath-hold, end-expiratory breath-hold, while performing a Valsalva manoeuvre (expiration while nose and mouth are closed) and while performing a Mueller manoeuvre (inspiration while nose and mouth are closed) [9]. The authors found that end-inspiratory breath-hold resulted in the second worst mean contrast enhancement within the pulmonary arteries compared to the other manoeuvres, only the Valsalva manoeuvre resulted in even worse contrast enhancement. A study by Chen et al. investigated possible reduction of TIC by performing CTPA in expiratory breath-hold [2]: here, CTPA in the expiratory phase could reduce the effect of TIC, however, only patients with indeterminate inspiratory CTPA were scanned in expiration, and the study did not demonstrate a general superiority of expiratory phase scanning for a general population.

A study by Renne et al. investigated the frequency of CTPA studies performed in end-inspiratory breath-hold with non-diagnostic image quality and if image quality in a second scan could be improved when allowing the patient to continue shallow breathing instead [13]: 0.9% of the CTPA studies were considered non-diagnostic and 0.5% of the studies were repeated with the alternative breathing command, all showing improved contrast in the pulmonary arteries. In this context it has to be mentioned that in our study cohort none of the patients was re-scanned immediately after the first scan, yet, applying the reported rate of 0.5% of repeated exams by Renne et al. to our study cohort would mean that 1/225 patients theoretically should have had a second scan due to non-diagnostic image quality. When comparing these small reported numbers of de-facto repeated scans to the frequency of TIC in the literature and in our study, further research efforts have to be undertaken on how to a-priori keep pulmonary contrast enhancement in CTPA studies routinely diagnostic, as to at least in our facility, a CTPA scan was not routinely repeated in case of reduced contrast enhancement in the pulmonary arteries.

After we received the study results, we changed the breathing command of the CTPA studies at our site to “end-expiratory breath-hold” and are planning a large randomised prospective trial where the reduction of TIC as one factor leading to reduced image quality and consecutively reduced diagnostic power of the CTPA exam will be further examined. One patient group will also perform a Mueller manoeuvre during their CTPA scan, as the latter breathing command resulted in the best mean intrapulmonary contrast enhancement in similar previous studies [9, 10].

Finally, a study published by Wang et al. in 2015 investigated possible improvement of CTPA contrast by means of right atrial monitoring combined with spontaneous respiration to trigger image acquisition and found that the technique provides optimal contrast enhancement in pulmonary arterial structures with minimal venous filling even with reduced doses of contrast material [14]. An important point to keep in mind with regard to the studies by Chen et al., Renne et al. and Wang et al. is that besides achievement of optimal contrast in the pulmonary vessels, the CTPA scan should be able to rule-in or rule-out potential differential diagnoses of pulmonary embolism such as pneumonia, congestive heart failure or aortic pathologies. Regarding studies with CTPA scans being acquired in end-expiratory breath-hold or even during shallow breathing, diagnostic assessment of such important differential diagnoses of pulmonary embolism in the emergency setting is given, while evaluation of the lung parenchyma with regard to e.g. interstitial lung diseases might be limited [6].

### Limitations

We did not further grade the TIC effect and subdivide patients into different groups of TIC severity, as data would not be sufficiently powered. What is more, we did not differentiate the effect of the presence of TIC on diagnostic accuracy, especially differentiating between contrast deterioration in central, segmental and sub-segmental segments. Assessment of other aetiologies of insufficient diagnostic image quality such as motion artefacts or higher image noise due to obesity etc. was also beyond the scope of this analysis.

Due to the retrospective nature of this study, the grade of compliance of the individual patient to follow breathing-commands was not monitored by measures such as registering flow curves via manometer attached to a mouthpiece or other [9, 10]. Especially, it was not possible to control whether a Valsalva manoeuvre—known to deteriorate contrast enhancement the most–was performed by the patient [8, 9].

Regarding the administration of i.v. contrast medium, an ordinary IDR of 1.4 gI/s and flow rate of 3.5 mL/s are used in our facility applied via a 20G i.v. catheter for CTPA studies. Yet, due to the retrospective character of this analysis and no flow curves being stored along with the image data in our PACS at the time, we do not have knowledge about the number of patients where flow rates probably would have to be reduced due to factors such as undersized i.v. catheters. It can be argued that flow rates have an effect on the incidence of TIC, albeit data has been published on sufficiently diagnostic CTPA image quality in 95% of patients at flow rates as low as 2.0 and 2.5 mL/s [15].

Finally, presence of a patent foramen ovale (PFO) is an often asymptomatic condition most people are unaware of. The bypass-effect from right to left ventricle via the PFO that increases in case of deep inspiration could further deteriorate contrast enhancement in the right heart and the pulmonary arteries. The prevalence of PFO in the general population is estimated to be at around 25 to 30% [4]. PFO prevalence in our study cohort is unknown, however, we did not observe a single patient with an aorto-pulmonary ratio >1 without TIC or incorrect bolus-tracking, consequently, the bypass effect—even if assumedly present in a certain percentage of our patients—did not lead to measurable effects.

## Conclusions

In this study it could be shown that TIC is a very common phenomenon occurring in more than every fifth patient that is undergoing a CTPA scan during inspiratory breath-holding after being told to inspire gently. TIC disproportionately often occurs in patients with lower absolute HU values in the pulmonary arteries and shows a significant negative correlation with increasing age.

## Supporting information

S1 Dataset PlosOne anonymized English headers. **Data for statistical analysis.** Qualitative and quantitative data of the 225 consecutive patients with CTPA scans screened and of the 222 patients included in the final analysis.
(XLSX)

## Abbreviations

TIC: transient interruption of contrast
CTPA: pulmonary computed tomography angiography
PA: pulmonary arteries
IVC: inferior vena cava
SVC: superior vena cava

## References

[1] Estrada YMRM, Oldham SA. CTPA as the gold standard for the diagnosis of pulmonary embolism. Int J Comput Assist Radiol Surg. 2011;6(4):557–63. 10.1007/s11548-010-0526-4 20689999

[2] Chen YH, Velayudhan V, Weltman DI, Balsam D, Patel N, Draves KA, et al Waiting to exhale: salvaging the nondiagnostic CT pulmonary angiogram by using expiratory imaging to improve contrast dynamics. Emergency radiology. 2008;15(3):161–9. 10.1007/s10140-007-0695-9 18189150

[3] Gosselin MV, Rassner UA, Thieszen SL, Phillips J, Oki A. Contrast dynamics during CT pulmonary angiogram: analysis of an inspiration associated artifact. Journal of thoracic imaging. 2004;19(1):1–7. 14712124 10.1097/00005382-200401000-00001

[4] Henk CB, Grampp S, Linnau KF, Thurnher MM, Czerny C, Herold CJ, et al Suspected pulmonary embolism: enhancement of pulmonary arteries at deep-inspiration CT angiography—influence of patent foramen ovale and atrial-septal defect. Radiology. 2003;226(3):749–55. 10.1148/radiol.2263012200 12601200

[5] Kuzo RS, Pooley RA, Crook JE, Heckman MG, Gerber TC. Measurement of caval blood flow with MRI during respiratory maneuvers: implications for vascular contrast opacification on pulmonary CT angiographic studies. AJR American journal of roentgenology. 2007;188(3):839–42. 10.2214/AJR.06.5035 17312076

[6] Mortimer AM, Singh RK, Hughes J, Greenwood R, Hamilton MC. Use of expiratory CT pulmonary angiography to reduce inspiration and breath-hold associated artefact: contrast dynamics and implications for scan protocol. Clinical radiology. 2011;66(12):1159–66. 10.1016/j.crad.2011.06.012 21889766

[7] Wittram C, Yoo AJ. Transient interruption of contrast on CT pulmonary angiography: proof of mechanism. Journal of thoracic imaging. 2007;22(2):125–9. 10.1097/01.rti.0000213566.78785.26 17527114

[8] Hamilton MC, Mortimer AM, Hughes J. Re: Use of expiratory CT pulmonary angiography to reduce inspiration and breath-hold associated artefact: contrast dynamics and implications for scan protocol. A reply. Clinical radiology. 2013;68(2):e99–100. 10.1016/j.crad.2012.10.015 23219308

[9] Gutzeit A, Froehlich JM, Walti S, Roos JE, Meissnitzer M, Hergan K, et al Suction/Inspiration against resistance or standardized Mueller maneuver: a new breathing technique to improve contrast density within the pulmonary artery: a pilot CT study. European radiology. 2015;25(11):3133–42. 10.1007/s00330-015-3735-y 26032878

[10] Gutzeit A, Roos JE, Hergan K, von Weymarn C, Walti S, Reischauer C, et al Suction against resistance: a new breathing technique to significantly improve the blood flow ratio of the superior and inferior vena cava. European radiology. 2014;24(12):3034–41. 10.1007/s00330-014-3328-1 25103533

[11] Ridge CA, McDermott S, Freyne BJ, Brennan DJ, Collins CD, Skehan SJ. Pulmonary embolism in pregnancy: comparison of pulmonary CT angiography and lung scintigraphy. AJR American journal of roentgenology. 2009;193(5):1223–7. 10.2214/AJR.09.2360 19843734

[12] Bernabe-Garcia JM, Garcia-Espasa C, Arenas-Jimenez J, Sanchez-Paya J, de la Hoz-Rosa J, Carreres-Polo JO. Has "respiratory coaching" before deep inspiration an impact on the incidence of transient contrast interruption during pulmonary CT angiography? Insights into imaging. 2012;3(5):505–11. 10.1007/s13244-012-0182-z 22773364 PMC3443274

[13] Renne J, Falck C, Ringe KI, Raatschen HJ, Wacker F, Shin HO. CT angiography for pulmonary embolism detection: the effect of breathing on pulmonary artery enhancement using a 64-row detector system. Acta radiologica (Stockholm, Sweden: 1987). 2014;55(8):932–7. 10.1177/0284185113507712 24103917

[14] Wang M, Li W, Lun-Hou D, Li J, Zhai R. Optimizing computed tomography pulmonary angiography using right atrium bolus monitoring combined with spontaneous respiration. European radiology. 2015;25(9):2541–6. 10.1007/s00330-015-3664-9 25850891

[15] Gossner J. Feasibility of computed tomography pulmonary angiography with low flow rates. Journal of clinical imaging science. 2012;2:57 10.4103/2156-7514.100999 23230539 PMC3515945

